# Caulk's Dental Annual for 1886-87

**Published:** 1887-02

**Authors:** 


Caulk's Dental Annual, No. 5, 1886-87.—This is a valuable *Annual* for reference, as it contains much useful information in the form of statistics relating to the principle matters connected with the science of dentistry. Colleges, Societies, Dental Laws, Patents, Members of State Dental Boards, and other useful information receive notice. Dr. L. D. Caulk, Editor and Publisher, Camden, Delaware.

